# Prevalence of Overweight and Obesity and Associated Diet-Related Behaviours and Habits in a Representative Sample of Adolescents in Greece

**DOI:** 10.3390/children9010119

**Published:** 2022-01-17

**Authors:** Rafaela Makri, Michail Katsoulis, Anastasios Fotiou, Eleftheria Kanavou, Myrto Stavrou, Clive Richardson, Afroditi Kanellopoulou, Philippos Orfanos, Vassiliki Benetou, Anna Kokkevi

**Affiliations:** 1 Department of Hygiene, Epidemiology and Medical Statistics, School of Medicine, National and Kapodistrian University of Athens, 115-27 Athens, Greece; rafamakri@med.uoa.gr (R.M.); phorfanos@med.uoa.gr (P.O.); 2 Institute of Health Informatics, Faculty of Population Health Sciences, University College London, London ΝW1 2DA, UK; m.katsoulis@ucl.ac.uk; 3 MRC Unit for Lifelong Health and Ageing, Institute of Cardiovascular Science, Faculty of Population Health Sciences, London WC1E 7HB, UK; 4 University Mental Health, Neurosciences, & Precision Medicine Research Institute “Costas Stefanis” (UMHRI), 115-27 Athens, Greece; afotiou@med.uoa.gr (A.F.); eleftheria.kanavou@gmail.com (E.K.); myrtostavrou@gmail.com (M.S.); akokkevi@med.uoa.gr (A.K.); 5 Department of Economic and Regional Development, Panteion University of Social and Political Sciences, 176-71 Athens, Greece; crichard@panteion.gr; 6 Department of Hygiene and Epidemiology, School of Medicine, University of Ioannina, 451-10 Ioannina, Greece; afkanellopoulou@gmail.com

**Keywords:** obesity, overweight, body weight, adolescents, body mass index, diet-related behaviours, dietary habits, cross-sectional study

## Abstract

Excessive body weight during adolescence represents a significant public health problem worldwide. Identifying factors associated with its development is crucial. We estimated the prevalence of overweight and obesity in a representative sample of 11, 13 and, 15-year-olds living in Greece and explored the association with diet-related behaviours and habits. Self-reported data on weight, height, diet-related behaviours and habits were used from 3816 students (1898 boys, 1918 girls) participants in the Greek arm of the international Health Behaviour in School-Aged Children (HBSC) study during 2018. Overweight and obesity were defined using the 2007 WHO growth charts classification. Prevalence of overweight was 19.4% in the total sample, 24.1% for boys and 14.7% for girls, and prevalence of obesity was 5.3% in the total sample, 7.3% for boys and 3.4% for girls, respectively. In the total sample, overweight (including obesity) was positively associated with male gender, low family affluence, skipping breakfast, and being on a diet, and inversely associated with age and being physically active. Eating rarely with the family was positively associated with overweight only among boys and eating snacks/meals in front of screens only among girls. No association was noted for eating in fast-food restaurants, consuming vegetables, fruits, sweets, and sugar-sweetened beverages.

## 1. Introduction

Over the years, excess body weight during childhood and adolescence has emerged as one of the most serious public health problems globally [1]. Childhood obesity has reached epidemic proportions, both in high and low-income countries, with the number of overweight and obese children having doubled or even tripled since 1970 [2,3]. According to the World Health Organization (WHO), in 2016, 18% of children and adolescents aged 5–19 years were overweight and obese worldwide, while significant geographical variations in obesity rates were noted [4]. Most importantly, overweight and obesity have important short and long-term adverse consequences on physical, mental, and emotional health of the child and future adult [1].

Studying the role of diet-related behaviours and dietary habits in the aetiology and prevention of overweight and obesity during childhood and adolescence has become increasingly important, since these behaviours and habits are formed early in life but also have the potential to be modified, although with difficulty, in the future. Furthermore, addressing obesity and losing weight during adulthood is much more challenging, especially after the age of 35 [5].

A number of diet-related behaviours which are highly prevalent among adolescents, such as skipping breakfast, eating infrequently with the family, eating in fast-food restaurants and in front of a screen, and dietary habits such as consuming sugar-sweetened beverages, have been studied in relation to overweight and obesity [6,7,8,9,10,11,12,13,14,15,16,17,18,19,20]. More specifically, skipping breakfast has been associated with increased body mass index (BMI) and a higher risk of becoming overweight or obese [6,8,18,19]. Having frequent family meals has been found to protect against the development of overweight and obesity among adolescents and 10 years later [9,10]. Snacking while watching TV has been reported to be positively associated with overweight [12,14,21], while similar associations, although less consistent, have been found for time spent in front of the computer or playing video games [11,13]. Frequent eating in fast-food restaurants has been associated with childhood obesity, mainly through the encouragement of unhealthy food options, although findings have not been consistent [15,16,20,22]. The consumption of sugar-sweetened beverages (SSBs) has been positively associated with obesity or unhealthy weight gain among children, with evidence drawn both from observational and interventional studies [17,23]. On the other hand, evidence on the role of sweets intake in relation to childhood obesity is not consistent. A recent meta-analysis concluded that sweets/confectionery consumption is inversely associated with overweight and obesity [24], whereas a significant negative correlation between the frequency of sweets intake and the likelihood of overweight during adolescence was found in 91% of 34 countries examined [13]. Daily consumption of a variety of vegetables and fruits constitutes an essential characteristic of a healthy diet. Nevertheless, the association of vegetable and fruit intake with overweight and obesity during childhood and adolescence is not strongly established [13,25,26,27]. A recent review concluded that the available data failed to support a protective role of high fruit and vegetable intake in relation to the risk of developing childhood obesity [28].

In Greece, overweight and obesity have reached epidemic proportions, to a greater extent than other European countries, both among children and adolescents [1,29,30,31,32,33,34,35]. With respect to adolescents, studies based on nationally representative samples are few, and the majority are not very recent [29,33,34,35,36,37,38]. During 2003, data from a representative sample of 14456 adolescents aged 13–19 years showed that overweight and obesity reached 29.4% in boys and 16.7% in girls [29], whereas in 2009, in data from 4786 children aged 10–12 years, overweight and obesity reached 42.8% among boys and 39.8% among girls [38]. In the context of the Health Behaviour in School-Aged Children (HBSC) survey, which provides a unique opportunity to monitor trends in obesity, prevalence of obesity among adolescents in Greece had doubled in 2014 (3.9%) in comparison to 2002 (2.0%), whereas a slight decrease, in overweight and obesity combined, in comparison to 2014 was evident especially among the 11-old-year girls in 2018 [35,39].

Based on the above, the aim of this study was to estimate the prevalence of overweight and obesity in a nationally representative sample of adolescents aged 11, 13, and 15 years old living in Greece during 2018 and to further explore its association with diet-related behaviours and habits with the ultimate goal of contributing to the development of evidence-based recommendations for the prevention and management of overweight and obesity in these age groups.

## 2. Materials and Methods

Data from the 2018 Greek arm of the HBSC study, conducted by the University Mental Health, Neurosciences, and Precision Medicine Research Institute every four years since 1998, were used. The HBSC study is a WHO multinational survey aiming to increase our knowledge of health and health-related behaviours among school-aged children [40]. The survey has been conducted at four-year intervals since 1985/86 in a growing number of countries following a commonly agreed international protocol developed by the study members. The instrument used to collect information for each survey consists of mandatory questions that all countries are required to include, optional questions on specific topic areas from which countries can choose, and country-specific questions related to issues of national importance. In Greece, the study received ethical approval from the Ministry of Education. A nationally representative sample of 11, 13, and 15-year-old students was selected using a multistage stratified random cluster sampling procedure, based on the HBSC study protocol, with the school class as the primary sampling unit. Stratification was based on (a) administrative region (NUTS II in the European Union’s classification; 10 out of 13 of Greece’s regions were covered, excluding the Ionian and North and South Aegean islands for logistical reasons), and (b) school type (comprehensive/technical/private) [41]. Active parental consent was required according to the study’s protocol, and prior to survey administration, students were informed orally that their participation was voluntary and anonymous and that they could opt-out of filling the survey. Questionnaires were administered in class by trained assistants during two consecutive regular class periods. Data were collected from 238 schools. For the present analyses, 8% of the completed questionnaires were excluded due to a high proportion of missing values or for being out of the age limits set by the HBSC study protocol. The final number of students participating in the 2018 survey was 3863 (1927 boys and 1936 girls).

### 2.1. Data on Anthropometry and Classification of Body Mass Index

Bodyweight (in kilograms) and height (in centimetres) without clothes and shoes, were based on self-reports. BMI was calculated as the ratio of weight in kilograms divided by the square of height in metres (kg/m^2^). Z-scores (a ΒΜΙ z score indicates how many units of the standard deviation a child’s BMI is above or below the average BMI value for his age group and gender) were then calculated and underweight, normal weight, overweight and obesity were defined using age- and gender-specific cut-offs recommended by the 2007 WHO growth charts (WHO) [42]. Underweight is defined as more than 2 standard deviations (SD) below the median, overweight is defined as more than 1 SD above the median to 2SD above the median, and obesity as more than 2SD above the median [42].

### 2.2. Diet-Related Behaviours and Food Consumption

Information related to diet-related behaviours and consumption of selected food groups and beverages were collected through standardised questionnaires.

Diet-related behavioural data was collected on the frequency of: (a) breakfast consumption (asked separately for schooldays and weekends), (b) eating snacks while watching TV or video, (c) eating snacks while sitting in front of a screen for homework or games, (d) eating meals while watching TV, (e) eating in fast-food restaurants and (f) eating meals with the family. With respect to breakfast consumption, the 5-day school week was preferred in this analysis to the 7-day week as the former was considered a better indicator of breakfast regularity. Additionally, the question about breakfast during weekends had many missing values. Specifically, students were asked to estimate how many schooldays during the week they had breakfast (defined as having more than one glass of milk or fruit juice), with responses ranging from “never” to “all five days”. Participants were further grouped according to their response into fewer categories: “never”, “1–4 days”, “always (5 days)” for eating breakfast. The possible responses for the questions on eating meals/snacks in front of TV/screen were six, ranging from “never” to “every day”. For the three behaviours related to eating snacks/meals in front of TV/screens (b–d) we further developed a combined score ranging from 0–6. The higher the score, the higher the frequency of eating in front of screens (TV/PC/Tablet). To determine the frequency of eating in fast-food restaurants, the available responses were seven, ranging from “never” to “five or more days/week”, and five with respect to the frequency of eating family meals, ranging from “every day” to “never”. Participants were further grouped according to their response into fewer categories: “never”, “less than once/month”, “1–3 days/month”, “weekly” for eating in fast-food restaurants, and “every day”, “almost every day”, and “rarely” for eating family meals.

Students’ weight-reduction behaviour measuring their attempts to lose weight was recorded with the following question, “At present are you on a diet or doing something else to lose weight?”. The possible answers were “No, my weight is fine”, “No, but I should lose some weight”, “No, because I need to put on weight”, and “Yes”. The question has been introduced in the international study since 1994 and has been validated in 2005 in Finland, showing acceptable test-retest reliability [43]. A dichotomous variable was further created to classify students according to whether or not they were on a diet “No”/“Yes”.

In relation to food consumption, students were asked to fill in a short food frequency questionnaire (FFQ) reporting their frequency of consumption during a typical week of the year for the following food groups and beverages: (a) fruits, (b) vegetables/salads, (c) sweets (candies, chocolates), (d) non-diet soft drinks and sugar-sweetened beverages. The possible responses for each group were seven and ranged from “never” to “more than once a day”. Students were further grouped according to their response into fewer categories: “less than once/week”, “1–4 days/week”, “5–6 days/week”, “every day” for fruit, vegetable, and sweet consumption, and “less than once/week”, “1–4 days/week”, “>5 days/week” for non-diet soft drinks and sugar-sweetened beverages intake. The short FFQ with the four mandatory items implemented in this analysis has been used since the 2001/2002 survey and validated in 2004/2005, showing acceptable reliability, although an overestimation of consumption frequencies was noted compared to a seven-day food diary [44,45].

### 2.3. Other Variables

Students were asked to report their gender, month, and year of birth. Age groups, age in months, and in years were computed. School-level data was also collected on the area where the schools were located. A family affluence scale (FAS), developed in the context of the HBSC international study, recorded students’ responses about material assets in their household [46,47,48]. More specifically, FAS was calculated using a six-item assessment of common material assets (having a family car, having own bedroom, number of bathrooms in the house, number of computers in the house, and having a dishwasher) or activities (frequency of family holidays abroad). Responses were scored and summed to form an HBSC FAS summary score, designated FAS-III, which has been shown to provide a valid indicator of relative affluence [47]. The affluence score is then used to identify groups of young people in the lowest 20% (low affluence), middle 60% (medium affluence), and highest 20% (high affluence) [39,48]. Physical activity was assessed by asking students to report the number of days over the past week during which they were physically active for a total of at least 60 min. Physical activity was defined as “any activity that increases your heart rate and makes you get out of breath some of the time”, with examples of such activities, assessing moderate to vigorous intensity physical activity. This item is used to identify those adolescents who meet the international guidelines for physical activity, which based on WHO recommendations, refer to at least an average of 60 min per day of moderate-to vigorous-intensity physical activity across the week [49]. Subjects were further grouped according to these response into fewer categories: “0–1 days”, “2–3 days”, “4–5 days”, and “6–7 days”.

### 2.4. Missing Values

Following an investigation of the extent of missing data on all variables included in the analysis (Figures S1 and S2), 4.6% missing values concerned BMI. Data are missing at random (MAR) when the probability that data are missing depends on the observed data. Multiple imputation is generally used if that assumption holds, as it provides a flexible and transparent means of imputing missing data. We conducted multiple imputation by age category (11, 13, and 15-year-olds), and we created multiple copies of datasets (*n* = 10) with imputed values. Imputation was stratified by region (18 clusters) (Table S1). We then calculated the BMI, the z-scores, and the BMI category. A flowchart depicting missing values in each variable and the final number of participants after the imputation process, as well as a similar flowchart with complete data are shown in Figures S1 and S2. The final sample of the study after the imputation was 3.816 adolescents, 1898 (49.7%) boys and 1918 (50.3%) girls.

### 2.5. Statistical Analysis

Participants’ data were summarized by frequencies for categorical variables, and by mean and standard deviation for continuous variables. Prevalence of underweight, normal weight, overweight, and obesity was estimated in the imputed data sample (*n* = 3816) as well as in the complete data sample (*n* = 3366), and we observed that prevalence in the various categories was practically the same (Table S2). Odds ratios (OR) and 95% confidence intervals (CI) derived from logistic regression models assessing the association between overweight status (overweight vs non-overweight), diet-related behaviours and food frequency intake were estimated. The overweight category included both overweight and obese students and the non-overweight included underweight and normal weight students.

A total of three models were calculated, the crude model (Model 1), a model where all variables entered in the model simultaneously adjusting for sex, age category, physical activity, and family affluence score (Model 2), and the fully adjusted model, where Model 2 was additionally adjusted for dieting (Model 3). We chose to construct this third model in order to be able to assess separately the influence of being on a diet or implementing any other weight reduction behaviour on the associations under study. The results from the crude model (Model 1) and the fully adjusted model (Model 3) are shown in the Tables of the main manuscript, whereas results for all three models are shown in the Tables S3–S5 in the supplementary material.

The significance level was defined at *p* < 0.05. Multicollinearity was assessed using the variance inflation factor (VIF) and no problem was detected (overall VIF = 1.11). All analyses took into account the survey design, i.e., geographically stratified sampling by regions (Nuts II) and cluster effect for school classes, via the svy commands of STATA. Analyses were performed using STATA 13.1 (STATA Corporation, College Station, TX, USA).

## 3. Results

Sociodemographic characteristics of the 3816 adolescents are presented in Table 1. The great majority were born in Greece (96.3%) while half of them were residing in two prefectures of Greece where the largest cities are situated, 37.4% in Attica (where the capital of Greece, Athens, is situated) and 14.6% in Thessaloniki (where the city of Thessaloniki is situated). Participants were evenly distributed across predefined age groups and grade at school by design. (Table 1).

Table 2 presents the percentages of underweight, normal weight, overweight and obese individuals among the 3816 adolescents (from imputed analysis) based on the 2007 WHO growth charts classification. The relevant percentages for the complete case analysis are presented in Table S2.

In the total sample, the prevalence of overweight was 19.4%; 24.1% among boys and 14.7% among girls, whereas the prevalence of obesity was 5.3%; 7.3% among boys and 3.4% among girls. Prevalence of underweight was 3.5% in the total sample, 3.2% among boys and 3.7% among girls. In the complete data sample (*n* = 3366), the prevalence of overweight was 19.2%; 24.2% among boys and 14.6% among girls, whereas the prevalence of obesity was 5.2% in the total sample, 6.9% among boys and 3.6% among girls. Prevalence of underweight was 2.9% in the total sample, 2.6% among boys and 3.3% among girls.

No statistically significant differences were observed between the proportion of overweight or obese by gender or age category both in imputed and in complete case analysis (Table S2).

Table 3 shows results from the crude (Model 1) and the fully adjusted model (Model 3) exploring the association between overweight, diet-related behaviours, and frequency of food consumption in the total sample. Table 4 presents results from the same analyses by gender.

Skipping breakfast was positively associated with being overweight in the total sample and among girls in the fully adjusted model. More specifically, those who never consumed breakfast on weekdays had 30% higher odds of being overweight compared to their counterparts who always ate breakfast (OR:1.30, 95% CI: 1.07–1.57), whereas the odds were even higher among girls that were skipping breakfast (OR:1.47, 95% CI: 1.09–1.98) (Table 3 and Table 4).

Eating rarely with the family was associated with being overweight only among boys (OR: 1.542, 95% CI: 1.05–1.91) in the fully adjusted model (Table 3 and Table 4)

A positive statistically significant association was found between the combined score of eating snacks/meals in front of screens and being overweight among girls. More specifically, one unit increase in the total score was associated with a 12% higher odds of being overweight in the fully adjusted model (OR: 1.12, 95% CI: 1.03–1.22) (Table 4). No statistically significant association was found in the total population and among boys.

No association was evident between the frequency of eating in fast-food restaurants and overweight in the total sample and by gender (Table 3 and Table 4).

Regarding intake of selected food and beverages, eating fruits less than 5 days per week compared to every day increased the odds of being overweight among boys (OR:1.52, 95% CI: 1.03–2.26) in the crude model, but the association was no longer significant in the fully adjusted model (Table 4). No association was evident between the frequency of vegetable intake and overweight in the total sample and by gender (Table 3 and Table 4). Εating sweets was not associated with obesity in the fully adjusted model (Table 3), although in the model that did not adjust for dieting, daily consumption of sweets compared to eating sweets less than once per week was associated with being overweight in the total sample (Model 2, OR:0.69, 95% CI: 0.51–0.94) and among boys (Model 2, OR:0.67, 95% CI: 0.46–0.99) (Tables S3 and S4). With respect to sugar-sweetened beverages, a moderate consumption (1–4 days per week) in comparison to the consumption of less than once per week (OR:1.20, 95% CI: 1.03–1.41) was associated with 20% higher odds of being overweight in the crude model but not in the fully adjusted (Table 3).

Being on a diet or having another weight reduction behaviour was strongly and statistically significantly positively associated with a 4-fold increase in odds of being overweight in the total sample (OR:4.47, 95% CI: 3.68–5.43), among boys (OR:4.32, 95% CI: 3.25–5.74), and among girls (OR:5.39, 95% CI: 4.00–7.25) in the fully adjusted models (Table 3 and Table 4).

Girls had 65% lower odds of being overweight compared to boys in the fully adjusted model (OR: 0.35, 95% CI: 0.29–0.42). Being overweight was negatively associated with age in the total sample and among girls, where 13-years-old and 15-years-old had lower odds of being overweight compared to 11-years-old in the fully adjusted model (Table 3 and Table 4). Lastly, the increased frequency of moderate to vigorous physical activity reported for the last 7 days was strongly and inversely associated with being overweight in the total sample and by gender (Table 3 and Table 4).

## 4. Discussion

In this representative sample of 11, 13, and 15-years-old adolescents living in Greece—almost 1 in 4 adolescents (24.7%)—were overweight/obese. Skipping breakfast in the total sample and among girls, rarely eating with the family among boys and frequently eating snacks/meals in front of screens among girls were all positively associated with being overweight (including obese). Being on a diet was positively associated with overweight in the total population and in both genders. Moreover, being a boy, having a low family affluence score, and being physically inactive were all associated with higher odds of being overweight. An inverse association was seen between age and overweight in the total sample and among girls. Eating in fast-food restaurants and intake of fruit, vegetables, sweets, and sugar-sweetened beverages were not associated with overweight.

The prevalence of overweight and obesity among adolescents in this study is in line with previously reported findings from regional, national and cross-national studies [29,32,33,36,37,50], although some have reported lower rates [34,35,51]. Differences in prevalence between studies may be attributed to differences in the age of study participants and differences in time periods, differences in the methodology used, such as the source of information on body weight and height, as well as the choice of different reference populations and cut-offs used to define overweight and obesity. In this study, we have used the 2007 WHO growth charts as a reference and the equivalent cut-off points to define overweight and obesity. The 2007 WHO growth reference for school-aged children and adolescents provides a suitable reference for the 5 to 19 years age group and is recommended by WHO for both clinical and epidemiological use. Currently, it is also used as the official growth reference for children and adolescents in Greece [35]. These findings can be used to evaluate the extent of the obesity problem in 11, 13, and 15-year old adolescents in Greece and reflect on the need to update existing and develop new programs and policies at the regional and national level in order to prevent and control overweight and obesity in the early years of life and beyond. Of note that the prevalence of underweight was quite low in this study sample.

Boys had a greater probability of being overweight compared to girls, which is in agreement with previous studies showing that the prevalence of obesity is greater among boys aged 5–19 years, especially in high and upper-middle-income countries worldwide [11,33,34,35,52,53,54,55]. Environmental and sociocultural factors, such as a greater tendency of boys to consume calorie-dense foods compared to girls [41,56,57,58,59], and more frequent weight-related concerns of girls compared to boys, are among the factors proposed as responsible for the observed difference. Furthermore, biological factors such as differences in body composition (e.g., girls in general have less fat-free mass, lower energy intake and fewer calorie needs than boys), and differences in hormone levels observed among girls, such as higher circulating concentrations of leptin, a hormone that suppresses appetite and promotes energy utilisation, may also be responsible [52,58,60,61,62].

Older adolescents were less likely to be overweight/obese in the total sample and among girls, as shown in other studies too [8,11,39]. It could be explained, at least partially, by the fact that as adolescents grow older, especially girls, they become more body-conscious and more vulnerable to sociocultural pressures conforming with specific body stereotypes [63].

Among diet-related behaviours examined, skipping breakfast was found to be positively associated with overweight in the total sample and among girls. Substantial evidence exists suggesting that breakfast consumption is negatively associated with overweight [11,18,19,50,64,65]. A recent narrative review highlighted the benefits of regular breakfast consumption on cardiorespiratory fitness, cardiovascular profile, cardiometabolic factors, and quality of life, and pointed out its protective role against childhood obesity [66]. Notably, a Swedish longitudinal study in adolescents with a 27-year follow-up showed that skipping breakfast in adolescence was an important predictor for the development of metabolic syndrome, central obesity, and high fasting glucose in adulthood. Breakfast skippers were found to have 2.18 times greater risk of central adiposity compared with breakfast consumers [7]. Additionally, eating breakfast once or less often during weekdays was also associated with poor diet quality in the Greek arm of the HBSC study [41].

A negative association was found between eating family meals and overweight, although only among boys. This finding is in line with previous studies suggesting that family meals are protective against the development of overweight and obesity during adolescence [9,10,50,67,68]. Family meals encourage social interaction between family members, the consumption of healthier food choices and higher quality foods, and parental dietary modeling as well as better control of the quality and quantity of a child’s meal [50,68,69].

Eating snacks or meals in front of screens (while watching TV, or while playing on a computer or tablet), measured by an overall index created in the context of this study combining all these behaviours, was not associated with overweight in the whole sample, although a positive statistically significant association was observed among girls. A number of studies have shown that sedentary behaviours such as spending time in front of screens can contribute to overweight, as well as to associated unhealthy snacking [12,14,21].

The frequency of eating in fast-food restaurants was not associated with overweight and obesity in this sample. This is in agreement with another study conducted in Greece using data from a large representative sample of children and adolescents aged 8–17 years during 2015 [16]. Similarly, French et al. did not find any association between the frequency of fast food consumption and overweight status [70]. Some other studies reported that the frequency of eating fast foods was associated with higher BMI, excessive fat in children, and elevated risk of childhood obesity [15,71], whereas other studies suggested that those associations are not easy to access due to many confounding factors [20].

Among the dietary variables examined, frequency of consumption of SSB was not associated with overweight status in the multivariate analysis, a result which is in line with the findings of an analysis based on the 2001–2002 HBSC study using data from 34 participating countries [13]. On the other hand, this finding is not in accordance with a substantial body of evidence linking regular consumption of SSB with a higher risk of obesity [17,23,26]. It should be noted that the HBSC questionnaire does not fully capture the range of sugary beverages available on the market, especially fruit juices and smoothies which are quite popular among adolescents. Current WHO guidelines concerning sugar intake for adults and children recommend reducing intake of free sugars to less than 10% of total energy intake. Sugar-sweetened beverages are among those beverages the regular consumption of which should be avoided [72,73].

No associations were observed between fruit and vegetable consumption and overweight in the current study. Many studies have shown similar findings [8,11,13,25,27], although not all [26]. A recent review by Newby on the role of plant-based diets and foods in the prevention of obesity concluded that available data on the role of fruit and vegetables specifically are inconsistent or generally null, and at the same time, have several methodological limitations [28]. Nevertheless, the lack of a consistent association with overweight and obesity does not justify any deviation from the recommendation to consume a variety of fruits and vegetables everyday, considering the beneficial role of this food group in the prevention of major chronic diseases such as cardiovascular diseases and certain malignancies.

It is interesting that although a significant negative relationship between overweight and sweets intake (candies and chocolates) was observed initially in the total sample, the association was no longer statistically significant in the fully adjusted model, which additionally controlled for weight-reduction behaviours. Being on a diet or implementing a weight reduction behavior is one of the possible explanations given for the reverse association of sweets intake with obesity often observed in relevant studies [13]. Underreporting of unhealthy food intake of overweight children compared to their non-overweight counterparts and lack of portion size information have also been proposed in order to explain this unexpected inverse association found in other studies.

Being on a diet or doing something else to lose weight was positively associated with overweight in both genders. Adolescents that reported being on a diet or doing something else to lose weight appeared to have four times higher odds of being overweight compared to the non-dieters. This is a particularly important finding showing that overweight children are subject to weight-reduction behaviors from the sensitive age of adolescence. Teasing, bullying, discrimination, and social exclusion of overweight or obese individuals may trigger an increased engagement with healthy eating and physical activity from a young age but also with an obsession with diet culture, which sometimes leads to eating disorders [74,75,76]. Body dissatisfaction and low self-esteem are also some of the psychosocial complications of childhood obesity that could also enhance a behaviour of dieting to lose weight [63]. Some adolescents also practiced unhealthy weight-control methods, such as diet pills or laxatives, vomiting, and smoking, which have been linked to obesity and eating disorders [77]. Information on the specific behaviour employed in this sample, such as the type of diet or the method to lose weight, was not available.

Regular physical activity of moderate to vigorous intensity was negatively associated with overweight in both girls and boys, a finding consistent with previous knowledge highlighting the importance of regular physical activity in the prevention and management of overweight and obesity throughout the lifetime [26,78,79,80].

Lastly, a negative association was evident between family affluence score and overweight, highlighting the importance of addressing social inequalities in overweight and obesity among adolescents in Greece [35]. Children of low family affluence may have limited opportunity for physical activity, lower quality of diet, and a misperception of ideal body weight. This is a consistent finding across the relevant studies [11,81,82,83].

Our study has several limitations. Its cross-sectional design does not allow us to infer causality for the observed associations. Secondly, all information retrieved, among them information on diet-related behaviors and diet habits as well as anthropometry, was self-reported, thus introducing a degree of information bias into the study. In particular, regarding the use of self-reported body weight and height, we assume that the prevalence of overweight has been underestimated compared to calculations based on actual height and weight measurements. This underestimation could be attributed to recall or social desirability bias and is generally greater among girls and as age and BMI values increase [35,84,85]. On the other hand, self-reported data of height and weight are commonly used in large epidemiological studies both to derive prevalence estimates and to identify valid relationships [86]. Furthermore, although the use of BMI alone for measuring obesity has its limitations, BMI has been widely acknowledged as a valid indirect measure of adiposity among children and adolescents worldwide [87,88]. Assessment of fruit, vegetable, sweets, and SSBs intake was based on a non-quantitative, self-reported food frequency questionnaire which did not allow a more detailed and in-depth study, also in terms of quantity, of the association of these food items with excess weight. In addition, no information was available for the study of other important food groups, such as meat, dairy, or cereals. Based on the results of the validation study of the short FFQ used, overestimation of the frequency of consumption of the studied items cannot be ruled out and should be taken into account in the interpretation of the findings. On the other hand, taking into account the overall structure and context of the HBSC survey and the relative limitations in time, space, and budget, only a limited number of food items, focusing on key indicators of adolescent diets, were possible. The score that was created in the context of this analysis for estimating the overall eating behaviour of adolescents in front of screens (TV, computer, or tablet) was not validated. Lastly, our analysis would have benefited more from an in-depth investigation of the possible mechanisms underlying the observed associations if less subjective data, such as biomarkers results, were also available.

Advantages of this study are the representative sample of adolescent participants, which allow us to generalize the prevalence of overweight and obesity to the general population of adolescents of the specific age groups living in Greece, the standardised international protocol of an established multinational survey, the use of WHO growth charts which also allows comparisons between countries, and the large sample size. An additional advantage is the performance of multiple imputation techniques in order to treat missing values (11.8% of the sample had missing values, among which 4.6% on BMI), which allowed us to minimise the possibility of selection bias and reduce the loss of valuable data caused by missing data.

## 5. Conclusions

In conclusion, in this representative sample of adolescents aged 11, 13, and 15 years old living in Greece, the prevalence of overweight and obesity was substantial, indicating that more action is urgently needed to address further this complex issue. Based on the findings of this study, promoting regular breakfast consumption, frequently eating with the family, and avoiding eating in front of the screens, would help to maintain a healthier body weight. The promotion of these diet-related behaviors could be incorporated in a multisectoral and multifactor action plan for the prevention and control of overweight and obesity, based on WHO recommendations, targeting children, adolescents, parents, teachers, and related health professionals both in the family and school setting as well as in the primary health setting. A special focus is further needed for younger age groups and adolescents from families of lower socioeconomic status. Differences between boys and girls concerning diet-related behaviors should also be considered and studied further in order to prevent and control effectively overweight and obesity.

## Figures and Tables

**Table 1:** Sociodemographic characteristics of 3816 participants in the 2018 Greek arm of the HBSC * study.

**Gender, *n* (%)**	
Boys	1898 (49.7%)
Girls	1918 (50.3%)
**Age Group, *n* (%)**	
11-years-old	1216 (31.9%)
13-years-old	1299 (34.0%)
15-years-old	1301 (34.1%)
**Region/municipality, *n* (%)**	
Attica	1427 (37.4%)
Thessaloniki	557 (14.6%)
Other	1832 (48.0%)
**Place of birth, *n* (%)**	
Greece	3675 (96.3%)
Other	141 (3.7%)
**Grade, *n* (%)**	
6th	1241 (32.5%)
8th	1307 (34.3%)
10th	1268 (33.2%)
**Family Affluence scale (FAS) score ^a^, *n* (%)**	
Low 20% affluence	545 (14.6%)
Middle 60% affluence	2392 (64.0%)
High 20% affluence	803 (21.4%)

^a^: Quantiles were calculated based on the FAS score distribution by gender and age group. * HBSC: Health Behaviour in School-aged Children study.

**Table 2:** Prevalence (%) of underweight, normal weight, overweight, and obesity (by gender and age category among 3816 participants in the 2018 Greek arm of the HBSC study *.

	Underweight (%)	Normalweight (%)	Overweight (%)	Obese (%)
All ages (*n* = 3816)				
Total	3.5%	71.8%	19.4%	5.3%
Boys	3.2%	65.4%	24.1%	7.3%
Girls	3.7%	78.2%	14.7%	3.4%
11-year-olds (*n* = 1216)				
Total	5.0%	67.7%	21.6%	5.7%
Boys	3.7%	63.0%	26.1%	7.2%
Girls	6.4%	72.3%	17.1%	4.2%
13-year-olds (*n* = 1299)				
Total	3.9%	71.3%	19.9%	4.9%
Boys	4.2%	66.2%	23.0%	6.6%
Girls	3.5%	76.3%	16.8%	3.4%
15-year-olds (*n* = 1301)				
Total	1.6%	76.1%	16.9%	5.4%
Boys	1.8%	66.7%	23.4%	8.1%
Girls	1.4%	85.6%	10.4%	2.6%

Abbreviations: HBSC study: Health Behaviour in School-Aged Children study, * Based on imputed data, Underweight: <−2SD, Overweight: >+1SD, Obesity: >+2SD as per 2007 WHO growth charts.

**Table 3:** Crude and adjusted Odds ratios (aOR) and associated 95% confidence intervals (CI) from logistic regression models exploring the association of Overweight with diet-related behaviours and habits among 3816 participants in the 2018 Greek arm of the HBSC study *.

ALL *n* = 3816	Crude Model		Fully Adjusted Model	
Variables	OR (95% CI)	*p*-Value	aOR (95% CI)	*p*-Value
Eating breakfast on weekdays				
Never	1.35 (1.14–1.59)	0.001	1.30 (1.07–1.57)	0.007
1–4 days	1.06 (0.87–1.28)	0.565	1.04 (0.85–1.28)	0.701
Always (5 days)	Ref.		Ref.	
Family meals				
Every day	Ref.		Ref.	
Almost every day	0.90 (0.76–1.08)	0.257	0.97 (0.81–1.17)	0.765
Rarely	1.19 (0.96–1.48)	0.113	1.22 (0.96–1.54)	0.098
Total score for behaviour of eating				
snacks/meals in front of screens (TV/PC/tablet)	1.04 (0.99–1.10)	0103	1.02 (0.96–1.08)	0.469
Eating in fast-food restaurants				
Never	Ref.		Ref.	
Less than once/month	1.14 (0.82–1.60)	0.430	1.29 (0.87–1.89)	0.203
1–3 days/month	0.96 (0.69–1.35)	0.831	1.07 (0.72–1.59)	0.751
Weekly	0.96 (0.67–1.37)	0.826	1.02 (0.67–1.57)	0.918
Fruits intake				
Less than once/week	1.31 (0.96–1.78)	0.088	1.17 (0.83–1.65)	0.356
1–4 days/week	1.16 (0.98–1.37)	0.075	1.09 (0.89–1.33)	0.413
5–6 days/week	1.00 (0.81–1.24)	0.987	0.93 (0.74–1.18)	0.564
Every day	Ref.		Ref.	
Vegetables intake				
Less than once/week	1.23 (0.95–1.59)	0.110	0.92 (0.69–1.22)	0.554
1–4 days/week	1.18 (0.90–1.31)	0.364	0.98 (0.80–1.21)	0.846
5–6 days/week	0.97 (0.79–1.20)	0.810	0.96 (0.77–1.19)	0.681
Every day	Ref.		Ref.	Ref.
Sweets intake				
Less than once/week	Ref.		Ref.	
1–4 days/week	0.89 (0.73–1.08)	0.245	1.02 (0.81–1.28)	0.873
5–6 days/week	0.86 (0.66–1.12)	0.270	1.12 (0.82–1.54)	0.473
Every day	0.68 (0.52–0.90)	0.008	0.84 (0.60–1.18)	0.312
Sugar-sweetened beverage intake				
Less than once/week	Ref.		Ref.	
1–4 days/week	1.20 (1.03–1.41)	0.022	1.08 (0.90–1.29)	0.416
>5 days/week	1.29 (0.98–1.70)	0.071	1.18 (0.86–1.62)	0.316
Being on a diet or doing something else to lose weight				
No	Ref.		Ref.	
Yes	3.33 (2.77–4.00)	<0.001	4.47 (3.68–5.43)	<0.001
Gender				
Boys	Ref.		Ref.	
Girls	0.48 (0.41–0. 57)	<0.001	0.35 (0.29-0.42)	<0.001
Age group				
11-year-olds	Ref.		Ref.	
13-year-olds	0.88 (0.73–1.06)	0.186	0.78 (0.63-0.97)	0.024
15-year-olds	0.76 (0.62–0.94)	0.009	0.62 (0.50-0.77)	<0.001
Family Affluence Scale (FAS)				
Low 20% affluence	Ref.		Ref.	
Middle 60% affluence	0.61 (0.50–0.75)	<0.001	0.61 (0.48-0.77)	<0.001
High 20% affluence	0.55 (0.42–0.71)	<0.001	0.57 (0.43-0.76)	<0.001
Physical activity (past 7 days)				
0–1 days	1.83 (1.40–2.38)	<0.001	2.53 (1.88-3.40)	<0.001
2–3 days	1.59 (1.29–1.97)	<0.001	1.98 (1.58-2.49)	<0.001
4–5 days	1.24 (0.99–1.54)	0.057	1.37 (1.08-1.73)	0.010
6–7 days	Ref.		Ref.	

Abbreviations: HBSC study, Health Behaviour in School-Aged Children study; aOR, adjusted odds ratio; CI, confidence interval. * Based on imputed data. Crude model: univariate model. Fully adjusted model: included all diet-related behaviours and dietary variables simultaneously adjusted for sociodemographic variables (sex, age category, FAS), physical activity and dieting.

**Table 4:** Crude and adjusted odds ratios (aOR) and associated 95% confidence intervals (CI) from logistic regression models exploring the association of overweight with diet-related behaviours and habits among 1898 school-aged boys and 1918 school-aged girls participants in the 2018 Greek arm of the HBSC study *.

	BOYS (*n* = 1898)				GIRLS (*n* = 1918)			
	Crude Model		Fully Adjusted Model		Crude Model		Fully Adjusted Model	
Variables	OR (95% CI)	*p*-Value	aOR (95% CI)	*P*-Value	OR (95% CI)	*p*-Value	aOR (95% CI)	*p*-Value
Eating breakfast on weekdays								
Never	1.38 (1.09–1.76)	0.008	1.22 (0.93–1.59)	0.150	1.48 (1.12–1.95)	0.006	1.47 (1.09–1.98)	0.011
1–4 days	1.01 (0.78–1.30)	0.960	0.94 (0.72–1.23)	0.639	1.30 (0.97–1.74)	0.083	1.22 (0.88–1.71)	0.235
Always (5 days)	Ref.		Ref.		Ref.		Ref.	
Family meals								
Every day	Ref.		Ref.		Ref.		Ref.	
Almost every day	0.97 (0.77–1.22)	0.807	1.02 (0.80–1.30)	0.884	0.93 (0.69–1.24)	0.602	0.89 (0.64–1.23)	0.464
Rarely	1.49 (1.14–1.96)	0.004	1.42 (1.05–1.91)	0.023	1.04 (0.76–1.43)	0.795	1.01 (0.71–1.44)	0.964
Total score for behaviour of Eating snacks/meals in front of screens (TV/PC/tablet)	0.96 (0.90–1.03)	0.224	0.96 (0.89–1.03)	0.276	1.11 (1.04–1.20)	0.003	1.12 (1.03–1.22)	0.011
Eating in fast-food restaurants								
Never	Ref.		Ref.		Ref.		Ref.	
Less than once/month	1.18 (0.75–1.86)	0.464	1.44 (0.87–2.39)	0.158	1.21 (0.67–2.17)	0.528	1.07 (0.56–2.03)	0.839
1–3 days/month	1.08 (0.69–1.67)	0.748	1.35 (0.81–2.25)	0.240	0.92 (0.53–1.61)	0.778	0.71 (0.38–1.32)	0.276
Weekly	0.96 (0.60–1.54)	0.871	1.23 (0.71–2.13)	0.457	0.91 (0.50–1.66)	0.764	0.71 (0.35–1.43)	0.340
Fruits intake								
Less than once/week	1.52 (1.03–2.26)	0.035	1.48 (0.93–2.35)	0.096	0.95 (0.53–1.69)	0.865	0.89 (0.48–1.65)	0.711
1–4 days/week	1.03 (0.81–1.30)	0.834	1.02 (0.77–1.35)	0.900	1.34 (1.02–1.75)	0.033	1.23 (0.90–1.68)	0.196
5–6 days/week	0.75 (0.57–0.99)	0.049	0.76 (0.56–1.05)	0.091	1.36 (0.95–1.94)	0.090	1.30 (0.89–1.90)	0.171
Every day	Ref.		Ref.		Ref.		Ref.	
Vegetables intake								
Less than once/week	1.05 (0.75–1.46)	0.795	0.81 (0.55–1.20)	0.303	1.19 (0.75–1.89)	0.456	1.13 (0.67–1.90)	0.658
1–4 days/week	0.90 (0.71–1.15)	0.397	0.83 (0.62–1.10)	0.185	1.24 (0.92–1.66)	0.155	1.25 (0.89–1.77)	0.200
5–6 days/week	0.80 (0.61–1.04)	0.098	0.81 (0.61–1.08)	0.147	1.15 (0.81–1.64)	0.419	1.26 (0.87–1.82)	0.227
Every day	Ref.		Ref.		Ref.		Ref.	
Sweets intake								
Less than once/week	Ref.		Ref.		Ref.		Ref.	
1–4 days/week	0.92 (0.72–1.17)	0.476	0.99 (0.75–1.31)	0.945	1.04 (0.73–1.48)	0.832	1.20 (0.79–1.82)	0.386
5–6 days/week	0.78 (0.56–1.09)	0.149	0.98 (0.67–1.45)	0.933	1.11 (0.71–1.74)	0.640	1.52 (0.89–2.61)	0.126
Every day	0.72 (0.51–1.02)	0.068	0.77 (0.50–1.17)	0.221	0.77 (0.49–1.20)	0.252	1.04 (0.62–1.74)	0.881
Sugar-sweetened beverage intake								
Less than once/week	Ref.		Ref.		Ref.		Ref.	
1–4 days/week	0.95 (0.77–1.18)	0.650	0.96 (0.76–1.21)	0.723	1.30 (1.01–1.66)	0.041	1.23 (0.93–1.64)	0.148
>5 days/week	1.08 (0.77–1.51)	0.648	1.18 (0.79–1.75)	0.423	1.20 (0.77–1.85)	0.415	1.07 (0.64–1.80)	0.784
Being on a diet or doing something else to lose weight								
No	Ref.		Ref.		Ref.		Ref.	
Yes	4.18 (3.18–5.48)	<0.001	4.32 (3.25–5.74)	<0.001	3.87 (2.94–5.09)	<0.001	5.39 (4.00–7.25)	<0.001
Age group								
11-year-olds	Ref.		Ref.		Ref.		Ref.	
13-year-olds	0.84 (0.65–1.08)	0.170	0.85 (0.63–1.13)	0.255	0.93 (0.71–1.23)	0.623	0.73 (0.54–0.98)	0.039
15-year-olds	0.92 (0.69–1.21)	0.549	0.86 (0.63–1.18)	0.356	0.55 (0.40–0.76)	<0.001	0.36 (0.26–0.52)	<0.001
Family Affluence scale (FAS)								
Low 20% affluence	Ref.		Ref.		Ref.		Ref.	
Middle 60% affluence	0.70 (0.54–0.92)	0.011	0.72 (0.54–0.98)	0.037	0.51 (0.37–0.70)	<0.001	0.50 (0.35–0.71)	<0.001
High 20% affluence	0.67 (0.49–0.91)	0.012	0.78 (0.55–1.11)	0.168	0.38 (0.25–0.58)	<0.001	0.35 (0.22–0.56)	<0.001
Physical activity (past 7 days)								
0–1 days	2.21 (1.59–3.05)	<0.001	2.20 (1.53–3.16)	<0.001	2.02 (1.30–3.14)	0.002	3.14 (1.86–5.28)	<0.001
2–3 days	2.06 (1.58–2.69)	<0.001	2.11 (1.60–2.78)	<0.001	1.71 (1.19–2.46)	0.004	1.89 (1.28–2.81)	0.002
4–5 days	1.26 (0.95–1.68)	0.105	1.33 (1.00–1.78)	0.051	1.48 (1.00–2.19)	0.052	1.58 (1.05–2.37)	0.029
6–7 days	Ref.		Ref.		Ref.		Ref.	

Abbreviations: HBSC study, Health Behaviour in School-Aged Children study; aOR, adjusted odds ratio; CI, confidence interval. * Based on imputed data, Crude model: univariate model, Fully adjusted model: included all diet-related behaviours and dietary variables simultaneously adjusted for sociodemographic variables (age category, FAS), physical activity and dieting.

## Data Availability

The dataset analysed in this study is available from the Epidemiology and Psychosocial Research Unit of the University Mental Health, Neurosciences, & Precision Medicine Research Institute (erevnaHBSC@epipsi.gr) on reasonable request.

## Supplementary Materials

The following supporting information can be downloaded at: https://www.mdpi.com/article/10.3390/children9010119/s1, Figure S1: Flowchart for complete case analysis examining the association between overweight/obesity and diet-related behaviours in the Greek-arm of the International Health Behaviour in School-aged Children (HBSC) study during 2018; Figure S2: Flowchart for imputed data analysis examining the association between overweight/obesity and diet-related behaviours in the Greek-arm of the International Health Behaviour in School-aged Children (HBSC) study during 2018; Table S1: Multiple Imputation Procedure; Table S2: Underweight, Normal weight, Overweight and Obesity prevalence (%) by gender and age category from complete case analysis of 3366 participants and from the analysis performed after imputation with the total 3816 participants in the 2018 Greek arm of the HBSC * study; Table S3: Odds ratios (OR) and associated 95% confidence intervals (CI) from logistic regression models exploring the association of Overweight with diet-related behaviours and habits among 3816 participants in the 2018 Greek arm of the HBSC study *. Table S4: Odds ratios (OR) and associated 95% confidence intervals (CI) from logistic regression models exploring the association of Overweight with diet-related behaviours and habits among 1898 school-aged boys participants in the 2018 Greek arm of the HBSC study *; Table S5: Odds ratios (OR) and associated 95% confidence intervals (CI) from logistic regression models exploring the association of Overweight with diet-related behaviours and habits among 1918 school-aged girls participants in the 2018 Greek arm of the HBSC study *.
