# Supplementary material for: Prevalence of Overweight and Obesity and Associated Diet-Related Behaviours and Habits in a Representative Sample of Adolescents in Greece
Source: Children (Basel). 2022 Jan 17;9(1):119. doi: 10.3390/children9010119 (PMC8774704; doi:10.3390/children9010119)
Supplement: Supplementary file 1 [file children-09-00119-s001.zip › children-1511830-supplementary.pdf]

Figure S1. Flowchart for complete case analysis examining the association between overweight/obesity and diet-related behaviours in the Greek-arm of the International Health Behaviour in School-aged Children (HBSC) study during 2018.

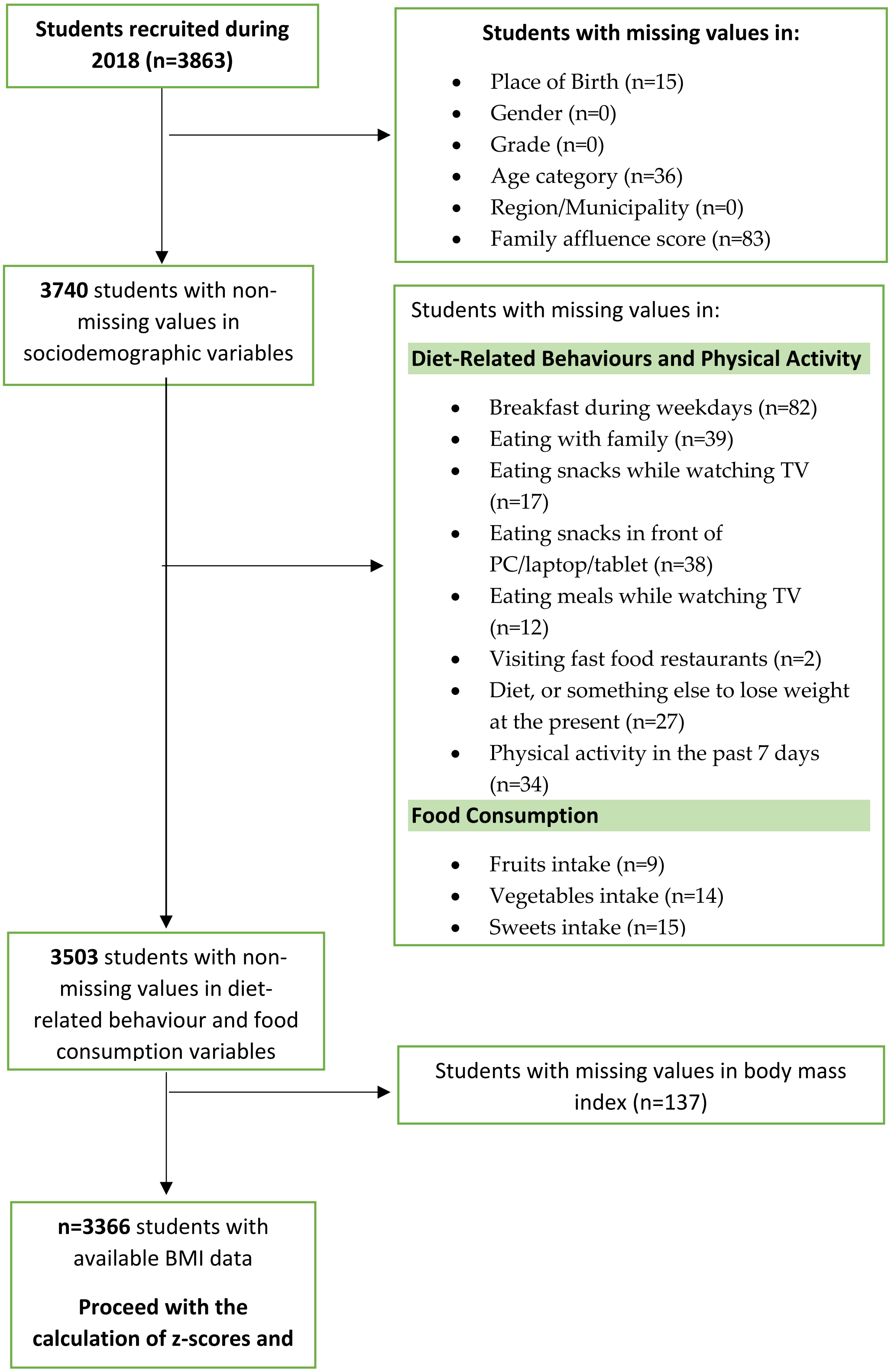

Figure S2. Flowchart for imputed data analysis examining the association between overweight/obesity and diet-related behaviours in the Greek-arm of the International Health Behaviour in School-aged Children (HBSC) study during 2018.

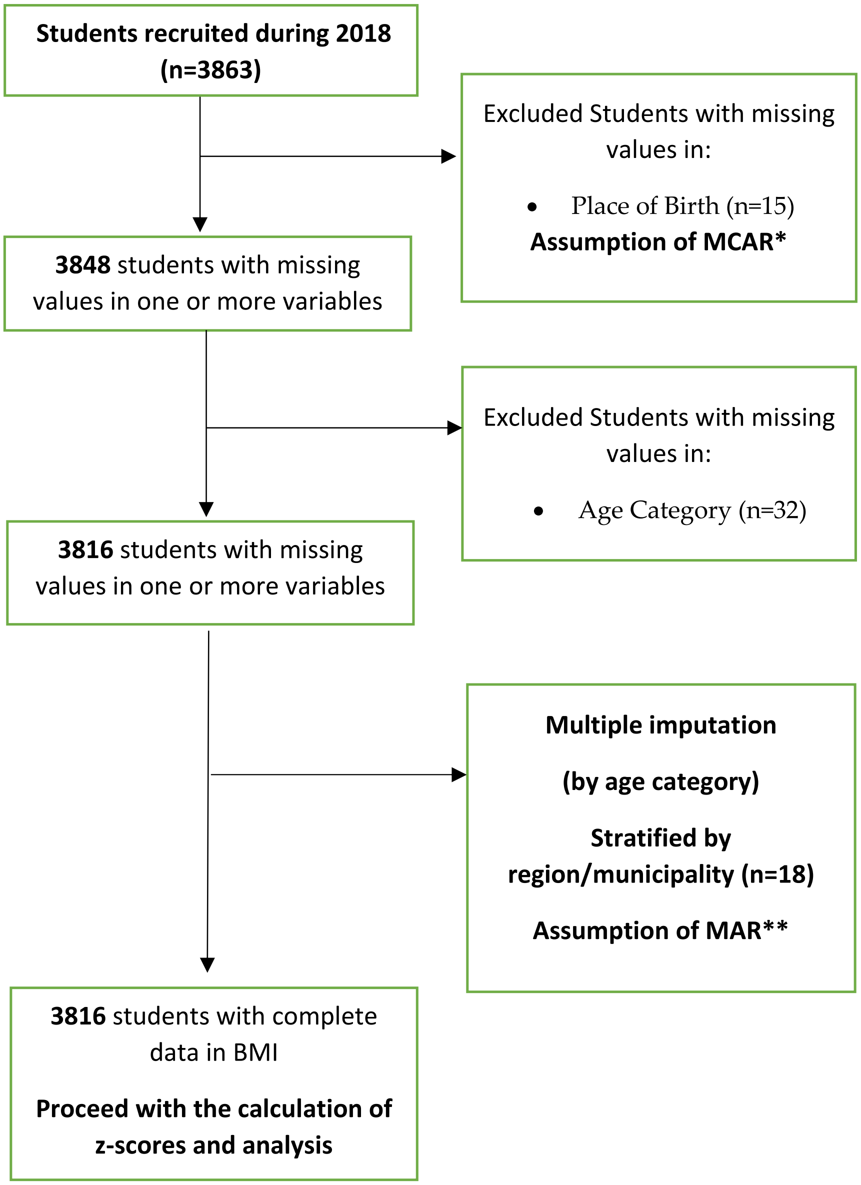

*MCAR: Missing completely at random

**MAR: Missing at random

Table S1. Multiple Imputation Procedure We created 10 copies of datasets in which missing values of bodyweight, bodyheight, breakfast consumption on weekdays, family meals, family affluence score, eating snacks while watching TV, eating snacks while working/playing on a computer, watching TV while having a meal, eating in fast food restaurants, being on a diet or something else to lose weight at the present, physical activity the past 7 days, eating fruits, eating vegetables, eating sweets, drinking coke/soft drinks were replaced by imputed values sampled from their predictive distribution, through multiple imputation by chained equations, which was applied separately in 3 age groups (11-, 13-, and 15-year-old), and we created multiple copies of datasets (n=10) with imputed values. Imputation was adjusted by region/ municipality (18 categories).The imputation model in each age group included the following variables that were mutually adjusted (plus, the sociodemographic variables gender (sex), region/municipality, and place of birth that had no missing data):

Variables	Modelled through
**Bodyweight**	predictive mean matching
**Bodyheight**	linear regression
**Breakfast consumption on weekdays**	multinomial logistic regression
**Family meals together**	multinomial logistic regression
**Family affluence score (FAS)**	multinomial logistic regression
**Eating snacks while watching TV**	multinomial logistic regression
**Eating snacks while work/play on computer**	multinomial logistic regression
**Watching TV while having a meal**	multinomial logistic regression
**Eating in fast food restaurants**	multinomial logistic regression
**Being on a diet or something else to lose weight at the present**	multinomial logistic regression
**Physical activity the past 7 days:**	multinomial logistic regression
**Eating fruits**	multinomial logistic regression
**Eating vegetables**	multinomial logistic regression
**Eating sweets**	multinomial logistic regression
**Drinking coke/soft drinks (SSBs)**	multinomial logistic regression

Table S2. Underweight, Normal weight, Overweight and Obesity prevalence (%) by gender and age category from complete case analysis of 3366 participants and from the analysis performed after imputation with the total 3816 participants in the 2018 Greek arm of the HBSC* study.

	**UNDERWEIGHT**		**NORMALWEIGHT**		**OVERWEIGHT**		**OBESE**	
	**Complete case**	**After imputation**	**Complete case**	**After imputation**	**Complete case**	**After imputation**	**Complete case**	**After imputation**
ALL AGES	**n=3366**	**n=3816**	**n=3366**	**n=3816**	**n=3366**	**n=3816**	**n=3366**	**n=3816**
TOTAL N(%)	**99 (2.9%)**	**3.5%**	**2446 (72.7%)**	**71.8%**	**647 (19.2%)**	**19.4%**	**174(5.2%)**	**5.3%**
BOYS	42 (2.6%)	3.2%	1075 (66.3%)	65.4%	392 (24.2%)	24.1%	111 (6.9%)	7.3%
GIRLS	57 (3.3%)	3.7%	1371 (78.5%)	78.2%	255 (14.6%)	14.7%	63 (3.6%)	3.4%
11-YEAR-OLDS	**n=1013**	**n=1216**	**n=1013**	**n=1216**	**n=1013**	**n=1216**	**n=1013**	**n=1216**
TOTAL	**43 (4.3%)**	**5.0%**	**695 (68.6%)**	**67.7%**	**218 (21.5%)**	**21.6%**	**57 (5.6%)**	**5.7%**
BOYS	15 (3.1%)	3.7%	314 (64.3%)	63.0%	127 (26.0%)	26.1%	32 (6.6%)	7.2%
GIRLS	28 (5.3%)	6.4%	381 (72.6%)	72.3%	91 (17.3%)	17.1%	25 (4.8%)	4.2%
13-YEAR-OLDS	**n=1173**	**n=1299**	**n=1173**	**n=1299**	**n=1173**	**n=1299**	**n=1173**	**n=1299**
TOTAL	**43 (3.6%)**	**3.9%**	**843 (71.9%)**	**71.3%**	**229 (19.5%)**	**19.9%**	**58 (5.0%)**	**4.9%**
BOYS	20 (3.6%)	4.2%	373 (67.0%)	66.2%	127 (22.8%)	23.0%	37 (6.6%)	6.6%
GIRLS	23 (3.7%)	3.5%	470 (80.0%)	76.3%	102 (16.6%)	16.8%	21 (3.4%)	3.4%
15-YEAR-OLDS	**n=1180**	**n=1301**	**n=1180**	**n=1301**	**n=1180**	**n=1301**	**n=1180**	**n=1301**
TOTAL	**13 (1.1%)**	**1.6%**	**908 (77.0%)**	**76.1%**	**200 (16.9%)**	**16.9%**	**59 (5.0%)**	**5.4%**
BOYS	7 (1.2%)	1.8%	388 (67.5%)	66.7%	138 (24.0%)	23.4%	42 (7.3%)	8.1%
GIRLS	6 (1.0%)	1.4%	520 (85.9%)	85.6%	62 (10.3%)	10.4%	17 (2.8%)	2.6%

* HBSC: Health Behaviour in School-aged Children study

Table S3. Odds ratios (OR) and associated 95% confidence intervals (CI) from logistic regression models exploring the association of Overweight with diet-related behaviours and habits among 3816 participants in the 2018 Greek arm of the HBSC study*.

	Model 1		Model 2		Model 3	
**Variables**	**OR (95% CI)**	**p-value**	**aOR[a] (95% CI)**	**p-value**	**aOR[b] (95% CI)**	**p-value**
**Eating breakfast on weekdays**						
Never	**1.35 (1.14-1.59)**	**0.001**	**1.31 (1.08-1.57)**	**0.005**	**1.30 (1.07-1.57)**	**0.007**
1-4 days	1.06 (0.87-1.28)	0.565	1.09 (0.89-1.33)	0.417	1.04 (0.85-1.28)	0.701
Always (5 days)	**Ref.**		**Ref.**		**Ref.**	
**Family meals**						
Every day	**Ref.**		**Ref.**		**Ref.**	
Almost every day	0.90 (0.76-1.08)	0.257	1.02 (0.85-1.22)	0.807	0.97 (0.81-1.17)	0.765
Rarely	1.19 (0.96-1.48)	0.113	**1.34 (1.07-1.67)**	**0.011**	1.22 (0.96-1.54)	0.098
**Total score for behaviour of eating snacks/meals in front of screens** (TV/PC/tablet)	1.04 (0.99-1.10)	0103	1.02 (0.97-1.08)	0.422	1.02 (0.96-1.08)	0.469
**Eating in fast-food restaurants**						
Never	**Ref.**		**Ref.**		**Ref.**	
Less than once/month	1.14 (0.82-1.60)	0.430	1.25 (0.87-1.80)	0.225	1.29 (0.87-1.89)	0.203
1-3 days/month	0.96 (0.69-1.35)	0.831	1.08 (0.75-1.56)	0.690	1.07 (0.72-1.59)	0.751
Weekly	0.96 (0.67-1.37)	0.826	1.04 (0.69-1.55)	0.861	1.02 (0.67-1.57)	0.918
**Fruits intake**						
Less than once/week	1.31 (0.96-1.78)	0.088	1.05 (0.75-1.46)	0.793	1.17 (0.83-1.65)	0.356
1-4 days/week	1.16 (0.98-1.37)	0.075	1.05 (0.86-1.27)	0.651	1.09 (0.89-1.33)	0.413
5-6 days/week	1.00 (0.81-1.24)	0.987	0.93 (0.74-1.16)	0.522	0.93 (0.74-1.18)	0.564
Every day	**Ref.**		**Ref.**		**Ref.**	
**Vegetables intake**						
Less than once/week	1.23 (0.95-1.59)	0.110	0.92 (0.70-1.22)	0.576	0.92 (0.69-1.22)	0.554
1-4 days/week	1.18 (0.90-1.31)	0.364	0.92 (0.75-1.12)	0.407	0.98 (0.80-1.21)	0.846
5-6 days/week	0.97 (0.79-1.20)	0.810	0.90 (0.73-1.12)	0.354	0.96 (0.77-1.19)	0.681
Every day	**Ref.**		**Ref.**		**Ref.**	**Ref.**
**Sweets intake**						
Less than once/week	**Ref.**		**Ref.**		**Ref.**	
1-4 days/week	0.89 (0.73-1.08)	0.245	0.98 (0.79-1.21)	0.826	1.02 (0.81-1.28)	0.873
5-6 days/week	0.86 (0.66-1.12)	0.270	0.92 (0.68-1.24)	0.580	1.12 (0.82-1.54)	0.473
Every day	**0.68 (0.52-0.90)**	**0.008**	**0.69 (0.51-0.94)**	**0.017**	0.84 (0.60-1.18)	0.312
**Sugar-sweetened beverage intake**						
Less than once/week	**Ref.**		**Ref.**		**Ref.**	
1-4 days/week	**1.20 (1.03-1.41)**	**0.022**	1.09 (0.91-1.29)	0.345	1.08 (0.90-1.29)	0.416
>5 days/week	1.29 (0.98-1.70)	0.071	1.17 (0.86-1.60)	0.323	1.18 (0.86-1.62)	0.316
**Being on a diet or doing something else to lose weight**						
No	**Ref.**				**Ref.**	**<0.001**
Yes	**3.33 (2.77-4.00)**	**<0.001**			**4.47 (3.68-5.43)**	
**Gender**						
Boys	**Ref.**		**Ref.**		**Ref.**	
Girls	**0.48 (0.41-0. 57)**	**<0.001**	**0.43 (0.36-0.51)**	**<0.001**	**0.35 (0.29-0.42)**	**<0.001**
**Age group**						
11-year-olds	**Ref.**		**Ref.**		**Ref.**	
13-year-olds	0.88 (0.73-1.06)	0.186	0.84 (0.68-1.02)	0.075	**0.78 (0.63-0.97)**	**0.024**
15-year-olds	**0.76 (0.62-0.94)**	**0.009**	**0.69 (0.57-0.85)**	**<0.001**	**0.62 (0.50-0.77)**	**<0.001**

**Family Affluence Scale (FAS)**						
Low 20% affluence	**Ref.**		**Ref.**		**Ref.**	
Middle 60% affluence	**0.61 (0.50-0.75)**	**<0.001**	**0.64 (0.51-0.79)**	**<0.001**	**0.61 (0.48-0.77)**	**<0.001**
High 20% affluence	**0.55 (0.42-0.71)**	**<0.001**	**0.59 (0.45-0.78)**	**<0.001**	**0.57 (0.43-0.76)**	**<0.001**
**Physical activity (past 7 days)**						
0-1 days	**1.83 (1.40-2.38)**	**<0.001**	**2.12 (1.61-2.81)**	**<0.001**	**2.53 (1.88-3.40)**	**<0.001**
2-3 days	**1.59 (1.29-1.97)**	**<0.001**	**1.89 (1.51-2.35)**	**<0.001**	**1.98 (1.58-2.49)**	**<0.001**
4-5 days	1.24 (0.99-1.54)	0.057	**1.33 (1.06-1.67)**	**0.014**	**1.37 (1.08-1.73)**	**0.010**
6-7 days	**Ref.**		**Ref.**		**Ref.**	

*Abbreviations: HBSC study: Health Behaviour in School-Aged Children study, aOR: adjusted odds ratio, CI: Confidence interval. *Based on imputed data, **Model 1**:unadjusted, **Model 2:** included all diet-related behaviours and dietary variables simultaneously adjusted for sociodemographic variables (sex,age category, family affluence score FAS), and physical activity (aOR[a]) **Model 3:** Model 2 including the variable dieting (aOR[b]).*

Table S4. Odds ratios (OR) and associated 95% confidence intervals (CI) from logistic regression models exploring the association of Overweight with diet-related behaviours and habits among 1898 school-aged boys participants in the 2018 Greek arm of the HBSC study*.

	Model 1		Model 2		Model 3	
**Variables**	**OR (95% CI)**	**p-value**	**aOR[a] (95% CI)**	**p-value**	**aOR[b] (95% CI)**	**p-value**
**Eating breakfast on weekdays**						
Never	**1.38 (1.09-1.76)**	**0.008**	1.24 (0.95-1.61)	0.112	1.22 (0.93-1.59)	0.150
1-4 days	1.01 (0.78-1.30)	0.960	0.98 (0.75-1.27)	0.864	0.94 (0.72-1.23)	0.639
Always (5 days)	**Ref.**		**Ref.**		**Ref.**	
**Family meals**						
Every day	**Ref.**		**Ref.**		**Ref.**	
Almost every day	0.97 (0.77-1.22)	0.807	1.05 (0.83-1.33)	0.687	1.02 (0.80-1.30)	0.884
Rarely	**1.49 (1.14-1.96)**	**0.004**	**1.54 (1.16-2.04)**	**0.003**	**1.42 (1.05-1.91)**	**0.023**
**Total score for behaviour of Eating snacks/meals in front of screens** (TV/PC/tablet)	0.96 (0.90-1.03)	0.224	0.97 (0.90-1.04)	0.341	0.96 (0.89-1.03)	0.276
**Eating in fast-food restaurants**						
Never	**Ref.**		**Ref.**		**Ref.**	
Less than once/month	1.18 (0.75-1.86)	0.464	1.30 (0.80-2.11)	0.283	1.44 (0.87-2.39)	0.158
1-3 days/month	1.08 (0.69-1.67)	0.748	1.26 (0.77-2.05)	0.355	1.35 (0.81-2.25)	0.240
Weekly	0.96 (0.60-1.54)	0.871	1.14 (0.67-1.94)	0.628	1.23 (0.71-2.13)	0.457
**Fruits intake**						
Less than once/week	**1.52 (1.03-2.26)**	**0.035**	1.28 (0.80-2.03)	0.298	1.48 (0.93-2.35)	0.096
1-4 days/week	1.03 (0.81-1.30)	0.834	0.98 (0.75-1.29)	0.898	1.02 (0.77-1.35)	0.900
5-6 days/week	**0.75 (0.57-0.99)**	**0.049**	0.77 (0.57-1.04)	0.087	0.76 (0.56-1.05)	0.091
Every day	**Ref.**		**Ref.**		**Ref.**	
**Vegetables intake**						
Less than once/week	1.05 (0.75-1.46)	0.795	0.83 (0.56-1.21)	0.331	0.81 (0.55-1.20)	0.303
1-4 days/week	0.90 (0.71-1.15)	0.397	0.82 (0.63-1.08)	0.155	0.83 (0.62-1.10)	0.185
5-6 days/week	0.80 (0.61-1.04)	0.098	0.77 (0.58-1.02)	0.066	0.81 (0.61-1.08)	0.147
Every day	**Ref.**		**Ref.**		**Ref.**	
**Sweets intake**						
Less than once/week	**Ref.**		**Ref.**		**Ref.**	
1-4 days/week	0.92 (0.72-1.17)	0.476	0.95 (0.73-1.24)	0.694	0.99 (0.75-1.31)	0.945
5-6 days/week	0.78 (0.56-1.09)	0.149	0.83 (0.57-1.19)	0.300	0.98 (0.67-1.45)	0.933
Every day	0.72 (0.51-1.02)	0.068	**0.67 (0.46-0.99)**	**0.046**	0.77 (0.50-1.17)	0.221

**Sugar-sweetened beverage intake**						
Less than once/week	**Ref.**		**Ref.**		**Ref.**	
1-4 days/week	0.95 (0.77-1.18)	0.650	0.98 (0.78-1.23)	0.851	0.96 (0.76-1.21)	0.723
>5 days/week	1.08 (0.77-1.51)	0.648	1.19 (0.81-1.76)	0.374	1.18 (0.79-1.75)	0.423
**Being on a diet or doing something else to lose weight**						
No	**Ref.**				**Ref.**	
Yes	**4.18 (3.18-5.48)**	**<0.001**			**4.32 (3.25-5.74)**	**<0.001**
**Age group**						
11-year-olds	**Ref.**		**Ref.**		**Ref.**	
13-year-olds	0.84 (0.65-1.08)	0.170	0.83 (0.64-1.09)	0.186	0.85 (0.63-1.13)	0.255
15-year-olds	0.92 (0.69-1.21)	0.549	0.86 (0.64-1.16)	0.337	0.86 (0.63-1.18)	0.356
**Family Affluence scale (FAS)**						
Low 20% affluence	**Ref.**		**Ref.**		**Ref.**	
Middle 60% affluence	**0.70 (0.54-0.92)**	**0.011**	0.75 (0.56-1.01)	0.054	**0.72 (0.54-0.98)**	**0.037**
High 20% affluence	**0.67 (0.49-0.91)**	**0.012**	0.77 (0.55-1.08)	0.129	0.78 (0.55-1.11)	0.168
**Physical activity (past 7 days)**						
0-1 days	**2.21 (1.59-3.05)**	**<0.001**	**2.08 (1.47-2.94)**	**<0.001**	**2.20 (1.53-3.16)**	**<0.001**
2-3 days	**2.06 (1.58-2.69)**	**<0.001**	**2.07 (1.58-2.72)**	**<0.001**	**2.11 (1.60-2.78)**	**<0.001**
4-5 days	1.26 (0.95-1.68)	0.105	1.27 (0.96-1.68)	0.091	1.33 (1.00-1.78)	0.051
6-7 days	**Ref.**		**Ref.**		**Ref.**	

*Abbreviations: HBSC study: Health Behaviour in School-Aged Children study, aOR: adjusted odds ratio, CI: Confidence interval. *Based on imputed data, **Model 1**: Crude, unadjusted, **Model 2:** included all diet-related behaviours and dietary variables simultaneously adjusted for sociodemographic variables (sex,age category, family affluence score FAS), and physical activity. **Model 3:** Model 2 including the variable dieting.*

Table S5. Odds ratios (OR) and associated 95% confidence intervals (CI) from logistic regression models exploring the association of Overweight with diet-related behaviours and habits among 1918 school-aged girls participants in the 2018 Greek arm of the HBSC study*.

	Model 1		Model 2		Model 3	
**Variables**	**OR (95% CI)**	**p-value**	**aOR[a] (95% CI)**	**p-value**	**aOR[b] (95% CI)**	**p-value**
**Eating breakfast on weekdays**						
Never	**1.48 (1.12-1.95)**	**0.006**	**1.45 (1.08-1.93)**	**0.013**	**1.47 (1.09-1.98)**	**0.011**
1-4 days	1.30 (0.97-1.74)	0.083	1.27 (0.93-1.74)	0.124	1.22 (0.88-1.71)	0.235
Always (5 days)	**Ref.**		**Ref.**		**Ref.**	
**Family meals**						
Every day	**Ref.**		**Ref.**		**Ref.**	
Almost every day	0.93 (0.69-1.24)	0.602	0.97 (0.70-1.33)	0.833	0.89 (0.64-1.23)	0.464
Rarely	1.04 (0.76-1.43)	0.795	1.13 (0.81-1.59)	0.478	1.01 (0.71-1.44)	0.964
Total score for behaviour of eating snacks/meals in front of screens (TV/PC/tablet)	**1.11 (1.04-1.20)**	**0.003**	**1.11 (1.03-1.21)**	**0.008**	**1.12 (1.03-1.22)**	**0.011**
**Eating in fast-food restaurants**						
Never	**Ref.**		**Ref.**		**Ref.**	
Less than once/month	1.21 (0.67-2.17)	0.528	1.18 (0.63-2.20)	0.601	1.07 (0.56-2.03)	0.839
1-3 days/month	0.92 (0.53-1.61)	0.778	0.85 (0.47-1.55)	0.594	0.71 (0.38-1.32)	0.276
Weekly	0.91 (0.50-1.66)	0.764	0.87 (0.45-1.68)	0.671	0.71 (0.35-1.43)	0.340
**Fruits intake**						
Less than once/week	0.95 (0.53-1.69)	0.865	0.79 (0.43-1.45)	0.439	0.89 (0.48-1.65)	0.711

1-4 days/week	**1.34 (1.02-1.75)**	**0.033**	1.18 (0.88-1.59)	0.270	1.23 (0.90-1.68)	0.196
5-6 days/week	1.36 (0.95-1.94)	0.090	1.28 (0.89-1.85)	0.185	1.30 (0.89-1.90)	0.171
Every day	**Ref.**		**Ref.**		**Ref.**	
**Vegetables intake**						
Less than once/week	1.19 (0.75-1.89)	0.456	1.12 (0.68-1.85)	0.657	1.13 (0.67-1.90)	0.658
1-4 days/week	1.24 (0.92-1.66)	0.155	1.09 (0.78-1.51)	0.621	1.25 (0.89-1.77)	0.200
5-6 days/week	1.15 (0.81-1.64)	0.419	1.16 (0.81-1.66)	0.426	1.26 (0.87-1.82)	0.227
Every day	**Ref.**		**Ref.**		**Ref.**	
**Sweets intake**						
Less than once/week	**Ref.**		**Ref.**		**Ref.**	
1-4 days/week	1.04 (0.73-1.48)	0.832	1.10 (0.75-1.61)	0.636	1.20 (0.79-1.82)	0.386
5-6 days/week	1.11 (0.71-1.74)	0.640	1.16 (0.71-1.90)	0.562	1.52 (0.89-2.61)	0.126
Every day	0.77 (0.49-1.20)	0.252	0.72 (0.44-1.18)	0.194	1.04 (0.62-1.74)	0.881
**Sugar-sweetened beverage intake**						
Less than once/week	**Ref.**		**Ref.**		**Ref.**	
1-4 days/week	**1.30 (1.01-1.66)**	**0.041**	1.23 (0.94-1.61)	0.127	1.23 (0.93-1.64)	0.148
>5 days/week	1.20 (0.77-1.85)	0.415	1.05 (0.65-1.70)	0.826	1.07 (0.64-1.80)	0.784
**Being on a diet or doing something else to lose weight**						
No	**Ref.**				**Ref.**	
Yes	**3.87 (2.94-5.09)**	**<0.001**			**5.39 (4.00-7.25)**	**<0.001**
**Age group**						
11-year-olds	**Ref.**		**Ref.**		**Ref.**	
13-year-olds	0.93 (0.71-1.23)	0.623	0.89 (0.67-1.18)	0.428	**0.73 (0.54-0.98)**	**0.039**
15-year-olds	**0.55 (0.40-0.76)**	**<0.001**	**0.50 (0.35-0.70)**	**<0.001**	**0.36 (0.26-0.52)**	**<0.001**
**Family Affluence scale (FAS)**						
Low 20% affluence	**Ref.**		**Ref.**		**Ref.**	
Middle 60% affluence	**0.51 (0.37-0.70)**	**<0.001**	**0.51 (0.37-0.71)**	**<0.001**	**0.50 (0.35-0.71)**	**<0.001**
High 20% affluence	**0.38 (0.25-0.58)**	**<0.001**	**0.39 (0.25-0.61)**	**<0.001**	**0.35 (0.22-0.56)**	**<0.001**
**Physical activity (past 7 days)**						
0-1 days	**2.02 (1.30-3.14)**	**0.002**	**2.14 (1.30-3.53)**	**0.003**	**3.14 (1.86-5.28)**	**<0.001**
2-3 days	**1.71 (1.19-2.46)**	**0.004**	**1.69 (1.15-2.47)**	**0.008**	**1.89 (1.28-2.81)**	**0.002**
4-5 days	1.48 (1.00-2.19)	0.052	**1.51 (1.01-2.26)**	**0.043**	**1.58 (1.05-2.37)**	**0.029**
6-7 days	**Ref.**		**Ref.**		**Ref.**	

*Abbreviations: HBSC study: Health Behaviour in School-Aged Children study, aOR: adjusted odds ratio, CI: Confidence interval. *Based on imputed data,* ***Model 1****: Crude, unadjusted,* ***Model 2:*** *included all diet-related behaviours and dietary variables simultaneously adjusted for sociodemographic variables (sex,age category, family affluence score FAS), and physical activity* ***Model 3:*** *Model 2 including the variable dieting.*
